# Nutrition and Frailty: Opportunities for Prevention and Treatment

**DOI:** 10.3390/nu13072349

**Published:** 2021-07-09

**Authors:** Mary Ni Lochlainn, Natalie J. Cox, Thomas Wilson, Richard P. G. Hayhoe, Sheena E. Ramsay, Antoneta Granic, Masoud Isanejad, Helen C. Roberts, Daisy Wilson, Carly Welch, Christopher Hurst, Janice L. Atkins, Nuno Mendonça, Katy Horner, Esme R. Tuttiett, Yvie Morgan, Phil Heslop, Elizabeth A. Williams, Claire J. Steves, Carolyn Greig, John Draper, Clare A. Corish, Ailsa Welch, Miles D. Witham, Avan A. Sayer, Sian Robinson

**Affiliations:** 1 Department of Twin Research and Genetics, King’s College London, St Thomas’ Hospital, Westminster Bridge Road, London SE1 7EH, UK; claire.j.steves@kcl.ac.uk; 2 Academic Geriatric Medicine, Faculty of Medicine, University of Southampton, Tremona Road, Southampton SO17 1BJ, UK; n.cox@soton.ac.uk (N.J.C.); h.c.roberts@soton.ac.uk (H.C.R.); 3 National Institute for Health Research (NIHR) Southampton Biomedical Research Centre, University of Southampton and University Hospital Southampton NHS Foundation Trust, Southampton SO16 6YD, UK; 4 Institute of Biological, Environmental and Rural Sciences, Aberystwyth University, Aberystwyth SY23 3DA, UK; tpw2@aber.ac.uk (T.W.); jhd@aber.ac.uk (J.D.); 5 Department of Epidemiology & Public Health, Norwich Medical School, University of East Anglia, Norwich NR4 7TJ, UK; r.hayhoe@uea.ac.uk (R.P.G.H.); a.welch@uea.ac.uk (A.W.); 6 School of Allied Health, Faculty of Health, Education, Medicine and Social Care, Anglia Ruskin University, Chelmsford CM1 1SQ, UK; 7 Population Health Sciences Institute, Newcastle University, Newcastle upon Tyne NE2 4AX, UK; sheena.ramsay@newcastle.ac.uk (S.E.R.); nuno.mendonca@nms.unl.pt (N.M.); 8 AGE Research Group, Translational and Clinical Research Institute, Newcastle University, Newcastle upon Tyne NE4 5PL, UK; antoneta.granic@newcastle.ac.uk (A.G.); christopher.hurst@newcastle.ac.uk (C.H.); philip.heslop@newcastle.ac.uk (P.H.); miles.witham@newcastle.ac.uk (M.D.W.); avan.sayer@newcastle.ac.uk (A.A.S.); 9 NIHR Newcastle Biomedical Research Centre, Newcastle upon Tyne Hospitals NHS Foundation Trust and Newcastle University, Newcastle upon Tyne NE4 5PL, UK; 10 Institute of Life Course and Medical Sciences, University of Liverpool, Liverpool L7 8TX, UK; m.isanejad@liverpool.ac.uk; 11 Institute of Inflammation and Ageing, College of Medical and Dental Sciences, University of Birmingham, Edgbaston, Birmingham B15 2TT, UK; d.v.wilson@bham.ac.uk (D.W.); c.welch@bham.ac.uk (C.W.); 12 MRC-Versus Arthritis Centre for Musculoskeletal Ageing Research, University of Birmingham and University of Nottingham, Birmingham B15 2TT, UK; c.a.greig@bham.ac.uk; 13 Epidemiology & Public Health Group, University of Exeter Medical School, Exeter EX1 2LU, UK; j.l.atkins@exeter.ac.uk; 14 EpiDoC Unit, CEDOC, NOVA Medical School, Universidade Nova de Lisboa, 1150-082 Lisbon, Portugal; 15 Comprehensive Health Research Centre (CHRC), NOVA Medical School, Universidade Nova de Lisboa, 1169-056 Lisbon, Portugal; 16 School of Public Health, Physiotherapy and Sport Science and UCD Institute of Food and Health, University College Dublin, Belfield, Dublin 4, Ireland; katy.horner@ucd.ie (K.H.); clare.corish@ucd.ie (C.A.C.); 17 The Medical Research Council Versus Arthritis Centre for Integrated Research into Musculoskeletal Ageing and The Department of Oncology and Metabolism, The University of Sheffield, Sheffield S10 2RX, UK; ertuttiett1@sheffield.ac.uk (E.R.T.); e.a.williams@sheffield.ac.uk (E.A.W.); 18 EDESIA PhD Programme, University of East Anglia Norwich Research Park, Norwich NR4 7TJ, UK; y.morgan@uea.ac.uk; 19 School of Sport, Exercise and Rehabilitation Sciences, University of Birmingham and NIHR Biomedical Research Centre, University Hospitals Birmingham NHS Foundation Trust and University of Birmingham, Birmingham B15 2TT, UK

**Keywords:** frailty, ageing, inflammation, appetite, microbiome, metabolome

## Abstract

Frailty is a syndrome of growing importance given the global ageing population. While frailty is a multifactorial process, poor nutritional status is considered a key contributor to its pathophysiology. As nutrition is a modifiable risk factor for frailty, strategies to prevent and treat frailty should consider dietary change. Observational evidence linking nutrition with frailty appears most robust for dietary quality: for example, dietary patterns such as the Mediterranean diet appear to be protective. In addition, research on specific foods, such as a higher consumption of fruit and vegetables and lower consumption of ultra-processed foods are consistent, with healthier profiles linked to lower frailty risk. Few dietary intervention studies have been conducted to date, although a growing number of trials that combine supplementation with exercise training suggest a multi-domain approach may be more effective. This review is based on an interdisciplinary workshop, held in November 2020, and synthesises current understanding of dietary influences on frailty, focusing on opportunities for prevention and treatment. Longer term prospective studies and well-designed trials are needed to determine the causal effects of nutrition on frailty risk and progression and how dietary change can be used to prevent and/or treat frailty in the future.

## 1. Introduction

**Figure 1:**
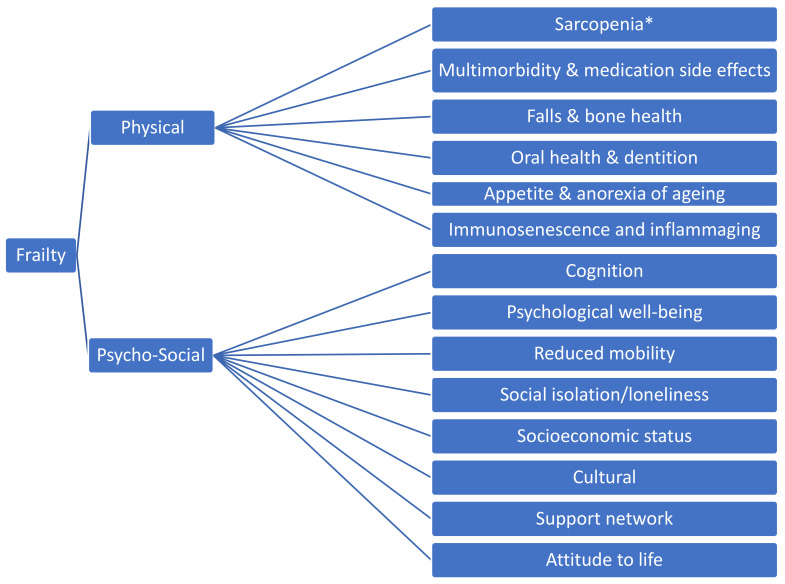
Components of Frailty * Sarcopenia is the age-related loss of skeletal muscle, quantified by objective measures of muscle strength, mass and function [6].

As populations around the world continue to age, the prevalence of age-related conditions rises in tandem [1,2]. Some of the leading causes of morbidity and mortality in older adults have been coined the ‘Geriatric Giants’ [3], and frailty is one of these. Frailty has been defined as an increased vulnerability to stressors across multiple bodily systems [4], a dynamic, multi-factorial process with cognitive and psychosocial components in addition to the physical components (Figure 1) [5]. Sarcopenia refers to a progressive loss of muscle mass and strength with age [6] and is considered another ‘Geriatric Giant’. While sarcopenia and frailty are distinct conditions, sarcopenia is considered to be the one of the key pathologies underpinning the syndrome of physical frailty [7].

The prevalence of frailty in older people has been estimated at 12–24% [8], with a wide range of estimates due to variation in definitions and diagnostic tools used. Inequality has been identified as a driver of frailty, particularly socioeconomic deprivation [9,10]. Frailty is associated with an increased risk of adverse outcomes, including falls, disability, hospitalisation, care home admission and death [4]. Unsurprisingly, it is associated with substantial increases in healthcare costs, with the Epidemiological study on chances of preventing, recognising early and optimally treating chronic diseases in an elderly population (ESTHER) study reporting that progression from non-frail (presence of 1–2 Fried phenotype criteria) to frail (presence of ≥3 Fried phenotype criteria) was associated a doubling of healthcare costs [11].

**Figure 2:**
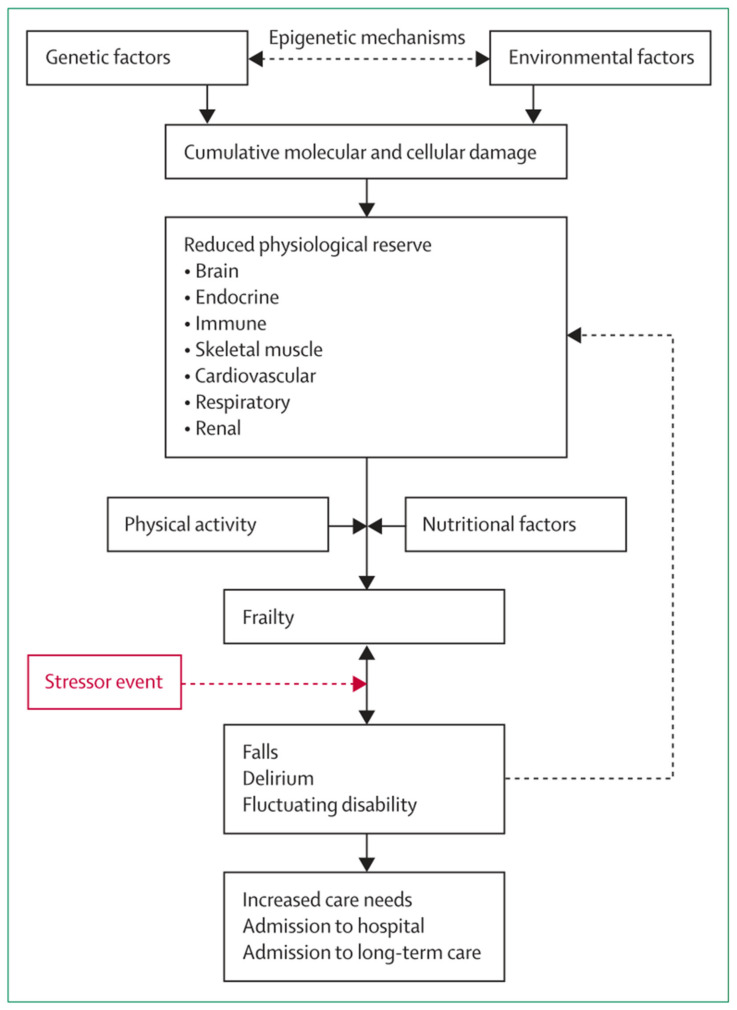
Schematic representation of the pathophysiology of frailty. Reproduced from [4]. Copyright permission has been obtained from [4].

In terms of pathophysiology, frailty is generally considered to be a complex multi-factorial process, as summarised in Figure 2, taken from the review by Clegg et al. [4]. Notably, nutritional factors are regarded as key components in this schema. Poor nutritional status has the potential to affect all five criteria used in Fried’s Frailty phenotype (weight loss, exhaustion, low physical activity, slow gait speed and weak grip strength) [12]; the concept of a vicious circle of declining energy utilisation is central to the Fried phenotype and has clear resonance with the field of nutrition. Other frailty measures include Rockwood’s Frailty Index [13], which is applied more in research settings, and the Clinical Frailty Scale [14], used commonly in clinical settings, among others, most of which take nutritional status into consideration to some degree [15,16].

Undernutrition, which refers to insufficient intake of energy and nutrients to meet an individual’s needs, is distinct from frailty; however, the two conditions overlap. Like frailty, undernutrition is associated with hospital admissions, length of hospital stay, readmission rates, general practitioner visits, prescriptions, admission to care homes, and dependency on others [17]. A high proportion of undernourished people are frail, and undernutrition leads to weight loss, which can contribute to the frailty syndrome [18]. At the other end of the spectrum is overnutrition; obesity increases frailty risk [19], with evidence that the risk of frailty increases with longer duration of obesity [20]. In terms of body composition, frailty has been associated with higher body fat mass and fat percentage, with low muscle mass and often no association with body mass index (BMI) [21,22,23,24]. This suggests that frailty may present with both underweight and overweight phenotypes, but consistently is associated with low muscle mass. However, apart from the effects of these differences in body composition arising from imbalances in energy intake, other aspects of nutrition such as diet quality and associated differences in nutrient intake are also likely to be important influences on frailty risk. For example, nutritional factors have direct and indirect effects on inflammatory processes and on oxidative stress, both of which are implicated in the aetiology of frailty [25,26]. Indeed, obesity is associated with chronic inflammation, with higher circulating inflammatory markers such as interleukin-6 (IL-6) [27].

Importantly, nutrition represents a modifiable risk factor for frailty, and as such, is a target for both prevention and treatment of this debilitating syndrome. However, to design effective dietary interventions, a clearer understanding of the key dietary components and underlying mechanisms of action are needed. Progress in understanding the role and importance of nutrition and to address how diet can be used for the prevention and treatment of frailty, requires a multi-disciplinary approach. This review evolved from a workshop held on 6 November 2020, which was attended by all authors. The workshop was one of a series funded by the Medical Research Council under the UK Nutrition Research Partnership (UK NRP) Call for Nutrition Hot Topic Workshops. It brought together a new group of experts from the fields of nutrition and frailty, from a variety of backgrounds, in order to promote dialogue to address the potential of diet within strategies to prevent and treat frailty. The workshop had two aims: firstly, to review the evidence and current understanding of the links between nutrition and frailty, and secondly, to consider the gaps in the evidence and identify unanswered questions.

## 2. Nutrition and Frailty—What Is the Evidence?

The following sections provide an overview of the current evidence for the links between nutrition and frailty. Overall, evidence is limited and there are challenges in collating findings across studies-leaving gaps in our understanding of the role and importance of nutrition as a modifier of frailty incidence and progression. Most of the evidence is observational, and much of this from cross-sectional studies. Importantly, cross-sectional evidence may be a particular challenge to understanding effects of nutrition on frailty, with suggestion of reverse causality in some studies, where living with frailty leads to changes in eating behaviour, rather than poor diet being its cause [28].

### 2.1. Dietary Patterns

There is increasing interest in the role of differences in diet and their influence on frailty risk. As reviewed in the following sections, this evidence is from observational studies; whole-diet intervention studies to alter dietary patterns are currently lacking.

#### 2.1.1. Diet Quality

A growing literature links variation in diet quality to frailty risk. Although there are differences in approaches to defining dietary patterns across studies, using *a priori* scoring methods, such as healthy eating indices or *a posteriori* approaches to identify dietary patterns, the overall findings appear similar: ‘healthier’ diets of higher quality, that are characterised by greater consumption of fruit, vegetables, and wholegrain foods, are associated with lower frailty risk. Indication of protective effects is found both in cross-sectional and longitudinal studies-and the effect size is large. For example, in a recent meta-analysis of fifteen studies (including cohort and cross-sectional data), the odds ratio for frailty associated with greater adherence to a healthy dietary pattern was 0.69 (95%CI 0.57–0.84). There was significant heterogeneity across studies, with differences in risk seen most clearly in studies from Mediterranean settings. Nine of the studies used Fried’s frailty phenotype or an adapted version [29]. More recent prospective cohort studies of diet quality from the US and UK, published since, also point to the beneficial effects of diets of higher quality.

In the US, Hengeveld and colleagues described associations between diet quality, assessed by the Healthy Eating Index, and 4-year incidence of frailty and prefrailty among older adults (age 70–81 years) in the Health ABC cohort: among non-frail and pre-frail participants at baseline, those with diets of poor/medium quality had a higher incidence of frailty over follow-up, even after adjusting for confounders including income, education, and smoking status [30]. Similarly, among women aged ≥60 years in the Nurses’ Health Study who were followed up over more than 22 years (diet quality defined in 4-yearly assessments using the alternative healthy eating index-2010), adherence to a healthy diet was associated with a lower risk of frailty, with a 1SD increase in diet quality score associated with a 10% reduction in risk, after adjustment for confounders including age, BMI, smoking status and energy intake [31].

Recent findings from the UK are consistent with these US studies: in a 3-year follow-up of older men in the British Regional Heart Study, those with a healthy (‘prudent’) dietary pattern were less likely to become frail (adjusted OR for prudent diet scores in top quarter of the distribution vs. bottom 0.53 (95% CI 0.30, 0.92), after adjustment for age, BMI, social class, region of residence, cardiovascular disease, smoking, alcohol consumption, and energy intake [32]. However, the way that diet quality is defined appears to be important; for example, in the UK study, there were no associations with Healthy Diet Indicator scores (based on World Health Organisation guidelines) and frailty risk [32]. Understanding the meaning of diet quality, and its implications for dietary exposures, will be essential to make progress in evaluating its effects, with the potential for useful insights into underlying mechanisms where diet quality is described in different ways within studies.

#### 2.1.2. Mediterranean Dietary Pattern

The meta-analysis of diet quality and frailty showed that beneficial effects of greater compliance with high quality dietary patterns were most marked in the sub-group of studies from Mediterranean countries [29]. The Mediterranean dietary pattern is characterised by high consumption of fruit, vegetables, and plant-based foods (legumes, nuts, seeds), use of olive oil, and lower consumption of meat and dairy foods, providing higher intakes of micronutrients, including antioxidant nutrients, as well as polyphenols and other plant bioactive compounds [33]. Linked to a range of beneficial effects on health [34,35], compliance with this pattern may also be relevant to risk of frailty [36].

Meta-analyses of the evidence on frailty risk show clear associations: across four longitudinal studies, greater adherence to the Mediterranean dietary pattern was associated with a lower risk of incident frailty, with a large effect size [37,38]. Two prospective studies from the US published since these reviews provide further confirmation of the benefits of the Mediterranean dietary pattern: greater compliance was associated with lower incident frailty among older women with type 2 diabetes in the Nurses’ Health Study [39], and among participants in the Osteoarthritis Initiative cohort [40], with both studies adjusting for age, sex, BMI, smoking status, energy intake, physical activity and co-mobidities. There are particular challenges in interpreting the differences in dietary exposures linked to the Mediterranean dietary pattern when collating evidence across studies [33,41]; for settings outside the Mediterranean, the findings from these recent US studies (based on adapted scoring methods) are therefore important.

#### 2.1.3. The Inflammatory Potential of Diet

The role of inflammation in the aetiology/pathophysiology of frailty has focused attention on the inflammatory potential of the diet. Specific dietary components that have anti-inflammatory actions, such as n-3 fatty acids, have been linked to protective effects [42]. However, more recent interest has been on the overall inflammatory potential of the diet, assessed from the balance of its pro-inflammatory and anti-inflammatory components [43]. Variation in this balance would be expected to have important implications for frailty as a number of studies have shown that pro-inflammatory diets, with higher Dietary Inflammatory Index (DII) scores, are associated with higher levels of circulating inflammatory markers such as C-reactive protein (CRP) and IL-6 [43,44].

Although the concept of dietary inflammatory potential is quite new, two recent systematic reviews were able to consider five studies [45,46]. Despite differences in design (mixed cross-sectional and longitudinal studies) and in the calculation of DII across studies, the findings were comparable: all showed that higher DII scores were associated with greater risk of frailty [45]. For example, in the longitudinal study of older adults (≥60 years) in the Study on Nutrition and Cardiovascular Risk (Seniors-ENRICA) cohort, who were followed up after three years, the odds ratio for frailty (adjusted for age, sex, education, energy intake, smoking status, BMI, diagnosed diseases, TV watching time and leisure-time physical activity) was 2.48 (95% CI: 1.42, 4.44), when comparing participants with baseline DII scores in the highest third of the distribution versus the lowest [47]. Other longitudinal analyses have suggested potential sex differences, with greater effects seen among men [48], although these have not been reported elsewhere. The size of reported effects and the overall consistency in findings across studies indicate the potential influence of pro-inflammatory diets on frailty. However, further studies are needed, particularly to consider the effects of the individual dietary components that determine DII scores [46] to fully understand their role and impact on frailty risk.

## 3. Foods

### 3.1. Fruit and Vegetables

As healthier dietary patterns are characterised by greater consumption of fruit and vegetables and associated with a lower risk of frailty, some studies have focused on their specific effects. Apart from their nutrient content, fruits and vegetables are sources of phytochemicals, such as polyphenols, that have anti-inflammatory and antioxidant actions [49]. Such protective effects could be important, as interventions to promote their consumption in older populations have been shown to be effective [50,51]. Unfortunately, the number of studies that examine their effects on frailty is relatively small.

In a systematic review published in 2018, Kojima and colleagues identified five prospective studies of community-dwelling older adults; follow-up periods ranged from 2 to 10.5 years [52]. Of these studies, three were judged to be of adequate quality, but only one had a primary focus on fruit and vegetable consumption. In this review, data from three prospective cohorts, with an average follow up period of 2.5 years were pooled; in fully adjusted models, a higher consumption of fruit and vegetables was associated with lower incident frailty, in a dose-response manner [53]. The same pattern was evident when fruit and vegetables were examined separately and when they were combined-and the effect size was large: OR for consumption of five or more portions of fruit and vegetables each day was 0.31 (0.13, 0.48) when compared to participants consuming up to one portion per day, even after adjusting for education and smoking status. These studies were from France and Spain, and the significance of these data for other settings is less clear.

Recent studies provide confirmatory evidence, based on cross-sectional data from mainland China [54], and longitudinal data from the US [55] and UK [56]. For example, in the English Longitudinal Study of Ageing (ELSA), after adjustment for age, sex, smoking, alcohol use, wealth, education, living alone, cognition, depressed mood, diabetes, and hyperlipidaemia, older adults without frailty at baseline who had a daily consumption of 5–10 portions of fruit and vegetables, had a lower risk of incident pre-frailty or frailty when followed-up over four years, although there were no differences among older adults who had very high fruit and vegetable consumption (>10 portions/d) [56].

Overall, the message from current evidence is that higher consumption of fruit and vegetables may have protective benefits. This potential benefit needs further exploration, particularly in countries such as the UK, where habitual consumption is low. For example, recent national survey data show that only 32% of men and women aged 65 to 74 years, and 19% of those over 75 years, meet recommendations to consume five or more portions of fruit and vegetables per day [57].

### 3.2. Dairy Foods

In comparison with fruit and vegetables, there have been fewer studies of milk and dairy foods in relation to frailty risk. Despite current interest in the effects of dairy foods on health, the evidence that links patterns and amounts consumed to differences in health outcomes is mixed [58]. Milk, however, is an important contributor to intakes of protein and other nutrients such as minerals, as well as a source of bioactive components that may have relevant protective effects on muscle health through anti-inflammatory, antioxidant, and other actions [59].

A systematic review published in 2019 identified only one prospective cohort study of dairy food consumption and frailty [58]. In this study of an older community-dwelling Spanish population, followed up over 3.5 years, greater consumption of low-fat milk and yogurt was associated with lower incidence of frailty, even in fully adjusted models, although there was no association with full-fat dairy product consumption [60]. More recently, positive effects of dairy consumption were reported in a prospective study of older adults in Japan: higher baseline consumption of milk and dairy products was associated with a lower risk of incident pre-frailty or frailty over a follow-up period of two years, after adjustment for age, sex and other covariates [61]. Overall, while some studies provide indications of protective effects, current understanding is limited by inadequate evidence for the effects of milk and dairy foods on frailty.

### 3.3. Ultra Processed Foods

Lately attention has focused on the effects of consuming ultra-processed foods (UPFs), defined according to the recent NOVA classification system [62]. Highly processed foods, such as meat products, savoury snack foods and sugar-sweetened beverages, make up a substantial part of diets in high-income settings and are typically energy-dense and low in nutrient content. These foods often characterise unhealthy dietary patterns and are linked to adverse effects on health, such as all-cause mortality, cardiovascular disease, metabolic syndrome, cancer, and others [63]. Notably, their effects on the gut microbiota are linked to pro-inflammatory processes that could be important in the pathophysiology of frailty [62].

To date, one prospective study has examined UPF consumption in relation to frailty. In an older Spanish population, Sandoval-Insausti and colleagues recently showed that participants with the highest consumption (top quartile of energy intake from UPF) had a three-fold increased risk of incident frailty over 3.5 years, when compared to those with low consumption (bottom quartile), even after adjustment for age, sex, education, smoking, alcohol intake, education, marital status, comorbidities, and medications [64]. Importantly, this association was robust to adjustment for confounding factors, that included lifestyle and co-morbidities. While further data are needed, this finding is consistent with the benefits of ‘healthier’ dietary patterns, and with current dietary recommendations that promote consumption of minimally processed foods.

## 4. Nutrients

### 4.1. Protein

There is considerable interest in the role of dietary protein as an influence on health in older age. The demonstration of anabolic resistance, that is the blunted muscle protein synthesis in response to protein consumption, has suggested the need to raise dietary protein recommendations in older age [65,66]. Such age-related changes are highly relevant to the maintenance of muscle mass and strength, and therefore to frailty-with insufficient protein intakes often implicated in its pathophysiology [67,68]. However, to date, much of the evidence has come from cross-sectional studies.

A systematic review and meta-analysis of observational studies published by Coelho-Júnior and colleagues in 2018 included ten studies; only three of these were longitudinal [69]. The meta-analysis, based on four cross-sectional studies, showed a lower frailty prevalence among older adults with high (compared with low) protein intakes, and the longitudinal evidence was largely consistent, with two of the three studies finding lower odds of incident frailty in older community-dwelling adults with higher protein intake [69]. However, the third study of a large US cohort of men aged ≥65 years, followed up over an average of 4.6 years, found that protein intake at baseline was not associated with the transition from robust status at baseline to intermediate or frail status at follow-up [70]. Newer longitudinal evidence published since, suggest that higher protein intakes have a protective effect. Specifically, lower incident frailty was observed among older Finnish women with higher protein intake at baseline [71], as well as among men and women (≥50 years) in the large Study on Health, Ageing and Retirement in Europe (SHARE) cohort [72], and among very old adults in the Newcastle 85+ study [73]. The three aforementioned studies adjusted for age, sex (other than in the female-only study) and a range of other potential confounders.

Putting the longitudinal studies together, the balance of evidence appears to favour beneficial effects of higher habitual protein intakes, linked to lower incident frailty across a range of populations, geography, and ages. Interventions to encourage consumption of protein-rich foods and/or dietary supplementation with additional protein should therefore be effective as part of strategies to prevent and/or manage frailty. However, there is limited evidence for the effectiveness of supplementation. For example, in a new systematic review and meta-analysis, Oktaviana and colleagues describe the effects of protein supplementation on functional frailty outcomes in pre-frail and frail older adults (≥65 years) [74]. Eight studies (503 participants) were reviewed; the outcomes were lean body mass and functional measures, that included grip strength and gait speed. The meta-analysis showed no effect of protein supplementation (up to 32 g/day), and no evidence of improvement in muscle mass, strength, or function. This may be due to many trials being carried out in participants who are replete in protein at baseline, or perhaps the variation in frailty screening tools used, the amount and type of protein supplementation, and the duration of the intervention.

More encouraging evidence has come from trials in which protein supplementation is combined with exercise training. In a meta-analysis of these trials, Liao and colleagues reviewed 22 studies of frail older adults: protein supplementation (including amino acid and enriched protein supplements) plus exercise training resulted in greater effects on outcomes such as lean body mass, hand grip and leg strength, when compared with exercise training alone [75]. While further evidence is needed, these findings may have important implications for the management of frailty in older adults, many of whom are likely to be inactive.

### 4.2. Antioxidant Nutrients

A number of studies link frailty and pre-frailty to higher oxidative stress [26]. For example, in a large European study of older adults, frail participants were shown to have lower blood concentrations of some antioxidant nutrients, including β-carotene and lycopene, when compared with non-frail participants, and higher concentration of oxidative stress biomarkers (protein carbonyls), after adjustment for age, sex, BMI, smoking status and season of blood sampling [76]. This highlights possible protective effects of dietary antioxidants, although current evidence is limited. There is some cross-sectional evidence; for example, frailty in older populations has been associated with lower intakes of antioxidant nutrients such as carotenoids and vitamin E [77], and with diets that have a lower total antioxidant capacity [78].

In addition, longitudinal evidence from the Women’s Health and Ageing Study, published more than a decade ago, pointed to the importance of carotenoid and vitamin E status, as higher serum concentrations predicted lower incident frailty over the following three years, despite adjustment for age, smoking status and other covariates [79]. However, there have been few longitudinal studies of antioxidant nutrients in the period since and evidence is inconsistent. The recent study by Das and colleagues that examined incident frailty over a three-year follow-up in a community-dwelling population of older men provides important new data [80]. Using an index of adequacy of dietary antioxidant intake (meeting ≥ 3 nutrient reference values for vitamin A, E, C, zinc vs. meeting ≤ 2), poor intake (particularly vitamin E) was related to a greater risk of incident frailty, after adjustment for 14 potential confounders. In contrast, a recent study, using data from four European cohorts, a micronutrient status pattern, characterised by higher serum carotenoid and alpha-tocopherol concentrations, was not associated with prevalent or with incident frailty [81]. The links between oxidative stress and frailty suggest protective effects of dietary antioxidants, but there are many gaps in current evidence. New data are needed, particularly from prospective cohort studies and randomised controlled trials.

### 4.3. Vitamin D

The proposed links between vitamin D status and muscle mass and strength have led to significant research activity in the fields of sarcopenia and frailty and compared with other aspects of nutrition, the evidence base for vitamin D and frailty is more established. The most recent meta-analysis of cross-sectional data included twelve studies; this showed that, in comparison with non-frail groups, vitamin D status (circulating 25(OH)D concentration) was lower in frail participants [82]. There are fewer longitudinal studies of vitamin D status and frailty, but systematic reviews of longitudinal data appear to be consistent, showing an association between lower status and greater risk of incident frailty [83].

Additionally, both cross-sectional and longitudinal studies provide evidence of dose effects [84]. Although this is a concern, as low vitamin D status is often common in older populations, there are few intervention studies to show whether vitamin D supplementation is effective in the prevention or treatment of frailty. Importantly, the first prospective cohort study to evaluate the effects of supplementation, reported by Bolzetta and colleagues in 2018, did not find evidence of protective effects; in a large study of older adults in the US, who were followed up over 8 years, incident frailty did not differ between supplement users and non-users [85]. The potential for reverse causality is a key consideration for observational studies, as older adults living with frailty may have less exposure to sunlight [86]. Intervention evidence is needed, but at present, the role of low vitamin D status remains uncertain.

## 5. Other Influences on Frailty Risk

### 5.1. Appetite

Appetite loss is common amongst older individuals, affecting approximately 20% of community dwelling individuals, rising in acute and long-term care settings [87]. Although an important symptom of medical illness or treatment, appetite loss can also be attributable to the ageing process in the anorexia of ageing [88]. The anorexia of ageing is a result of changes to physiological, hedonic, environmental, and social influences on appetite in later life [88]. It is an important cause of reductions in not only overall energy intake but also dietary diversity, including a decline in protein consumption [89,90]. Poor appetite in older individuals is associated with undernutrition and frailty predominantly through weight loss [91,92,93]. Therefore, identification and targeted intervention for individuals with poor appetite may prove effective in preventing or delaying onset of frailty. However, evidence for effective interventions targeting the anorexia of ageing is scarce [94]. In addition, many foods associated with reduced frailty, e.g., high protein foods, are also highly satiating [95]. Reassuringly, a recent meta-analysis of 22 studies involving 857 participants found that while appetite ratings were suppressed in some studies of acute protein supplementation, there is either a positive effect or no effect on total energy intake in acute and longitudinal studies, respectively [96]. Exploring potentially modifiable influences on appetite, such as mood, physical activity, and interaction with the gut microbiota, may provide fruitful avenues to producing interventions for the anorexia of ageing. This should be done alongside attention to management strategies to maintain adequate energy and nutrient intake, which mitigate for poor appetite. Person-centred approaches based on an understanding of individuals’ perceptions of appetite loss, which appear to follow distinct narratives relating to either feelings of excessive satiety or negative psychological responses to food and eating, are likely to be of most value in terms of engagement and compliance.

### 5.2. Oral Health

Oral health problems constitute a major burden of morbidity [97], and the risk of developing oral ill-health increases substantially with age [98]. Recent trends show older people are retaining teeth for longer, bringing challenges of increased susceptibility to dental diseases [99]. Examples of poor oral health in older adults include tooth loss, periodontal disease, xerostomia (dry mouth), and dental caries and associated complications e.g., infection, root caries [100]. Multiple oral conditions can co-occur. The consequences of these conditions include difficulty speaking, swallowing and/or eating, reduced quality of life and an increased risk of morbidity and mortality [100,101]. Oral ill-health can also impact adversely on dietary habits and nutritional intake [102,103,104]; oral conditions are associated with poor diet quality, inadequate intake of micronutrients, protein and fruit and vegetables.

Poor oral health and frailty co-occur in older people, especially those in long term care [101,105]. A bi-directional association between the two may be likely, as longitudinal studies have suggested oral ill health could contribute to the development of frailty, and increased risk of incident frailty has been observed with a number of the oral health conditions, particularly tooth loss and dry mouth [105,106,107]. Oral ill-health and frailty share pathophysiological pathways and aetiological factors including chronic disease, poor self-care, smoking, BMI, socioeconomic factors etc. [100,101]. Furthermore, they also share potential mediators, including nutrition, dietary deficiencies, and inflammation. [100,101,108].

Older people with frailty are more vulnerable to dental disease and may have worse access to dental treatment. This oral ill health may further worsen frailty, highlighting the need for primary and secondary prevention of oral diseases in older adults, as well as improved proactive management of oral health in frail older adults and those at risk of developing frailty.

## 6. Can Dietary Change Be Used to Prevent and Treat Frailty?

As reviewed above, much of the current evidence on the role of nutrition in the prevention and/or treatment of frailty is observational, with many studies based on cross-sectional data. There are far fewer intervention studies that can provide the evidence of causal effects which is needed to inform our understanding of the potential of diet to be used as a strategy to influence the development and progression of frailty [28]. Apart from the limited evidence base, there are challenges in understanding existing interventional studies. Firstly, many studies have used multi-pronged interventions, making it difficult to dissect out the role of nutrition, with many trials using a combined exercise and nutrition approach. Secondly, the supplements and foods used often contain a range of nutrients, limiting understanding of the effects of individual components. Thirdly, there are additional challenges in terms of how frailty is measured [109]. Variation in measures used across studies can make comparisons difficult. Often the primary outcome is one component of the frailty phenotype, rather than reduction in overall frailty.

### 6.1. Nutritional Interventions for Frailty

To date, most dietary intervention studies have combined nutritional supplementation with exercise training. For example, in the systematic review by Apóstolo and colleagues, of 33 interventions considered, three were of supplementation alone [110]. One of the reviewed studies by Ng et al. (2015) used an oral nutritional supplement designed to increase energy intake by 20% and to provide supplementary iron, calcium, folate, vitamins B6, B12 and D to an older population of pre-frail and frail adults for a 6-month period. The study involved parallel intervention groups, one of which was the nutritional supplement and another of which was a combination intervention which included the supplement, alongside cognitive and physical training [111]. Both groups had a reduction in Fried’s frailty score sustained at 12 months, but the effect was greater in the combination group, even after adjustment for compliance. No age or sex differences were reported.

Others have focused on more specific nutritional interventions. For example, included in the systematic review [110] Kim et al. conducted a trial looking at the effects of Milk Fat Globule Membrane (MFGM) supplementation for a 3-month period as a treatment for frailty and found a reduction in frailty status in those who received the supplement, but a greater reduction in those who received MFGM plus exercise, after adjusting for age and baseline frailty score [112].

Overall, there is little evidence from dietary intervention studies to inform clinical guidance [113], although consistent with other multidomain trials, there may be more positive messages from studies where supplementation is combined with exercise training [114]. However, the quality of this limited evidence is low, both for studies of supplementation alone, and for studies of combinations which included supplementation [110]. Furthermore, there is little current information to evaluate separate nutritional influences on development vs. progression of frailty. The potential of diet to prevent or treat frailty is therefore not yet clear. Further data are needed to build the evidence to inform the design of effective dietary intervention strategies, which consider challenges specific to ageing adults, ensuring strategies are appropriate and acceptable to those who will actually be affected by them: older people with, or at risk of developing, frailty.

### 6.2. Looking Forward

Important advances are being made in related fields, which have the potential to influence frailty research. For example, our understanding of the gut microbiome, which refers to the bacteria, archaea, viruses, and eukaryotic microbes that reside inside the gut, has expanded exponentially in recent years. More recent again, is the study of the ‘metabolome’ which measures metabolites in blood or other biological samples. The ability to measure these host factors, has led to the suggestion of a move away from the current one-size-fits-all approach to nutritional recommendations, towards a more personalised approach [115]. For example, the recent Personalised Responses to Dietary Composition Trial (PREDICT) study reported dramatic variations in dietary inflammation after meals within healthy adults, a cohort which included identical twins [116]. Both the gut microbiome and the metabolome have the potential to play a key role in improving understanding of the role of diet and its influence on frailty risk.

#### 6.2.1. The Gut Microbiome

The gut microbiome has a collective genome size that may be as much as 150-fold that of the human host [117], and some have argued that it merits consideration as an organ of the human body in its own right, with intrinsic functions and metabolic needs [118]. The composition of the gut microbiome is heavily influenced by diet, with particular food types showing predictable shifts in the host’s bacteria [119]. Acute states such as inflammation or a severe stress such as a burn injury can also lead to changes in the gut microbiota [118,120]. In addition, the composition of an individual’s gut microbiota can influence how food eaten is processed. For example, the gut microbiota have been proposed to play a key role in anabolic resistance of skeletal muscle to dietary protein [121], and therefore in the development of sarcopenia and physical frailty. Despite this bidirectional relationship, the microbiome composition is typically stable in adults and shifts secondary to a dietary change tend not to be sustained once that dietary intervention is removed [122].

Notably, the resilience of the gut microbiome is reduced in frailty, and it can become more vulnerable to changes in lifestyle, medications, and disease [123,124,125,126]. People living with frailty often have concurrent sarcopenia, chronic disease and/or polypharmacy, each of which may also influence, and potentially are influenced by, the gut microbiota [127]. More research is needed to ascertain the causative role, if any, of the gut microbiome in the development of a frailty syndrome.

Ghosh et al. (2019) profiled the gut microbiota of >1200 frail and non-frail participants aged 65–79 years across Europe before and after a 12-month dietary intervention (New dietary strategies addressing the specific needs of elderly population for healthy ageing in Europe: NU-AGE diet), a Mediterranean-style diet. Specific microbiome alterations were reported, which were positively associated with markers of reduced frailty (measured using Fried score), improved cognition, and negatively associated with inflammatory markers including CRP and interleukin-17, even after adjustment for a range of potential confounders [128]. Further studies are needed to identify whether the microbiome changes are driver of, or consequences of, the change in frailty status in trials such as this one.

A randomised controlled trial of a prebiotic (fructooligosaccharides) supplement for 13 weeks in sixty frail adults aged 65 years or older reported significant improvements in two frailty criteria, exhaustion (fatigue score; pre1.4 ± 1.7 post0.8 ± 1.4, *p* < 0.05) and handgrip strength (pre10.6 ± 8.2, post12.4 ± 3.2, *p* < 0.05), and also a reduction in overall frailty index score (pre0.22 ± 0.09, post0.20 ± 0.08, *p* < 0.001), versus those given placebo [129,130]. While there are no studies to our knowledge testing other prebiotic supplements, or probiotic supplements for reduction of frailty, there is some limited evidence that such supplements may improve other age-related conditions that may contribute to overall frailty, such as cognition and bone health, and this has been summarised recently by Jayanama et al. [131]. The crucial point is that the gut microbiome is potentially *malleable*, and can be targeted using dietary changes, prebiotics, probiotics, and even faecal microbial transplants, thus representing an exciting therapeutic target for preventing and treating frailty.

#### 6.2.2. Metabolome

Metabolomics or metabolomic profiling involves the simultaneous measurement of metabolites in order to characterise a biological system [132]. Metabolomics is a useful tool in the analysis of complex biological systems, as the metabolome is the link between an individual’s genotype and phenotype [133]. Pujos-Guillot et al. (2019) used metabolomics to elucidate the metabolites in serum which are associated with pre-frailty sub-phenotypes in older people [132]. Identification of circulating pre-frailty molecular biomarkers is vital to understand the trajectory of pre-frailty and frailty and to develop early warning predictors of decline, prior to observed physiological differences. The serum metabolome has been the main target of investigation within frail and pre-frail individuals. Rattray et al. (2019) concluded that dysregulation in the carnitine shuttle and subsequent alterations in energy metabolism are associated with a clinical presentation of frailty [134]. Investigations into the muscle metabolome in frail individuals have also been able to elucidate key metabolic processes, such as branched chain amino acid catabolism, but highlighted the dissimilarity between skeletal muscle metabolites and those circulating in plasma [135].

Metabolomics has been extensively used to measure the dietary intake of individuals and predict dietary patterns within populations through the use of biomarkers of food intake, found in urine [136,137]. Recent technological advances have facilitated community collection of urine [138], which combined with metabolomics, presents a potential solution to passively monitor nutritional status and dietary intake of individuals within the community. Objective reporting of dietary intake, through analysis of urinary biomarkers [139,140] has not been exploited in pre-frail and frail populations; but could be a useful tool for monitoring habitual dietary intake going forward.

## 7. Discussion

There is a growing body of evidence that links variation in habitual diets and nutritional status to frailty risk in older populations. Some of the most robust evidence is for higher quality or ‘healthier’ diets, particularly a Mediterranean dietary pattern, with an indication of protective effects for some of the foods that characterise these patterns, (such as greater consumption of fruits and vegetables and lower consumption of ultra-processed foods), as well as associated nutrients (such as higher intakes of protein and antioxidant nutrients). These observational studies are consistent with potential biological mechanisms, with evidence of dose-response effects in some studies. The evidence linking frailty with the inflammatory potential of diet, vitamin D status or with intake of dairy foods remains insufficient to draw any conclusions; however, initial studies are suggestive that links may exist, and this warrants further study. There are many gaps in understanding, particularly the lack of whole-diet intervention studies in which frailty is the primary outcome [141]. This limits translation of current evidence and our evaluation of how diet might be used in strategies to protect the health of older populations.

A host of personal factors and inequalities affect variations in diet among older people and may confound observational studies. Examples include appetite and oral health, as discussed above, but also socioeconomic status, education, availability of food, and physical activity, as well as age-related changes such as sense of smell, taste, and gastric emptying. While most of the cited studies in our review did adjust for age, sex, BMI, smoking, energy intake and co-morbidites, there was significant variation in what confounding variables were included. Very few, if any, adjusted for income, socioeconomic status, alcohol intake, psychological well-being, loneliness, social network, race, or geographical location. Ideally these should be considered carefully when designing future randomised controlled trials in this field of enquiry.

We chose to focus on the existing evidence linking nutrition and frailty. It was outside the scope of this review to consider what the best approaches are to achieve lasting dietary changes, or what the barriers and potential solutions are to improve nutritional status in older people. Whether research findings should be accepted into dietary recommendations and clinical practice, and how best to implement such changes is also outside of the scope of this paper; however, it warrants mentioning as a crucial factor to be considered in future research. It should be noted that the narrative nature of this review is a limitation, which was due to its origins, in a workshop bringing together experts from frailty and nutrition backgrounds.

## 8. Conclusions

More work is needed in understanding how dietary modification impacts on the fundamental biological processes thought to underpin frailty (oxidative stress, inflammation, microbiome, etc.). Novel methodological techniques including multi-omics approaches, such as metabolomics are needed to drive progress in this area. There is also a need for further investigation into more personalised approaches to ameliorate frailty with nutrition, particularly as evidence grows that many individuals have different responses to the same nutrient, including identical twins [116]. Lastly, longer term prospective studies and well-designed interventions are needed, which involve older people in their design, to determine the causal effects of nutrition on frailty risk and progression and how diet can be used to prevent, delay and/or treat frailty in the future.

## Data Availability

Not applicable.
